# Supplementary material for: Knockdown of Mitogen-Activated Protein Kinase Kinase 3 Negatively Regulates Hepatitis A Virus Replication
Source: Int J Mol Sci. 2021 Jul 10;22(14):7420. doi: 10.3390/ijms22147420 (PMC8303476; doi:10.3390/ijms22147420)
Supplement: Supplementary file 1 [file ijms-22-07420-s001.zip › ijms-1233563-Supplementary Materials.pdf]

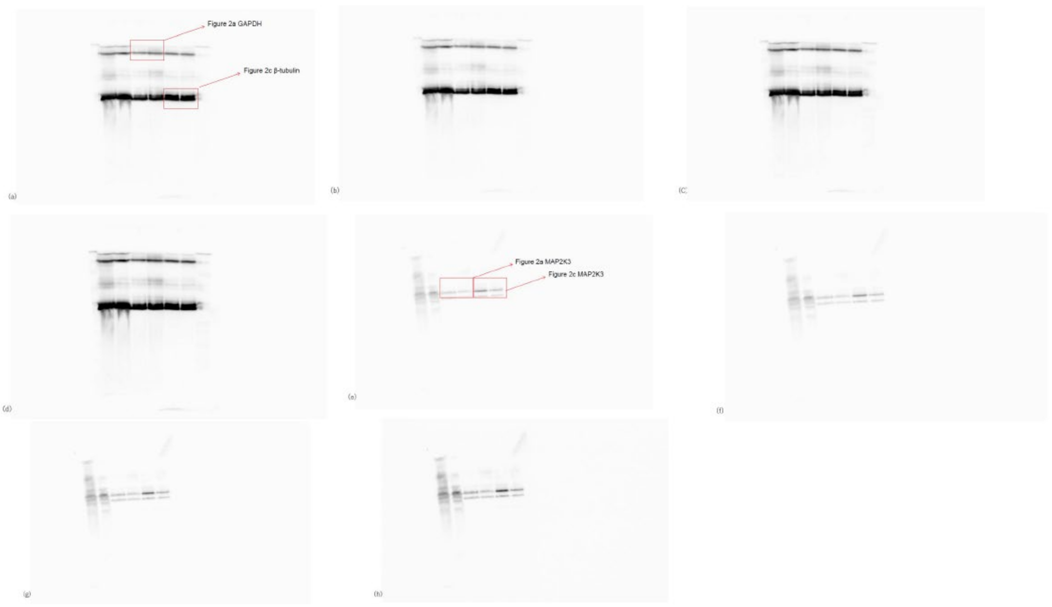

Supplementary Materials. (a)-(e): The original image for the blots [Figure 2 (a) and 2 (c)] used for the analysis of Figure 2 (b) and 2 (d). These data are expressed as the means ± standard deviations of triplicate determinations from one experiment which was representative of three independent experiments.
